# Geostatistical model of the spatial distribution of arsenic in groundwaters in Gujarat State, India

**DOI:** 10.1007/s10653-020-00655-7

**Published:** 2020-07-11

**Authors:** Ruohan Wu, Joel Podgorski, Michael Berg, David A. Polya

**Affiliations:** 1 Department of Earth and Environmental Sciences, School of Natural Sciences and Williamson Research Centre for Molecular Environmental Science, University of Manchester, Manchester, M13 9PL UK; 2 Eawag, Swiss Federal Institute of Aquatic Science and Technology, 8600 Dübendorf, Switzerland

**Keywords:** Groundwater, Arsenic, Health impacts, Gujarat, Logistic regression, Geostatistics

## Abstract

Geogenic arsenic contamination in groundwaters poses a severe health risk to hundreds of millions of people globally. Notwithstanding the particular risks to exposed populations in the Indian sub-continent, at the time of writing, there was a paucity of geostatistically based models of the spatial distribution of groundwater hazard in India. In this study, we used logistic regression models of secondary groundwater arsenic data with research-informed secondary soil, climate and topographic variables as principal predictors generate hazard and risk maps of groundwater arsenic at a resolution of 1 km across Gujarat State. By combining models based on different arsenic concentrations, we have generated a pseudo-contour map of groundwater arsenic concentrations, which indicates greater arsenic hazard (> 10 μg/L) in the northwest, northeast and south-east parts of Kachchh District as well as northwest and southwest Banas Kantha District. The total number of people living in areas in Gujarat with groundwater arsenic concentration exceeding 10 μg/L is estimated to be around 122,000, of which we estimate approximately 49,000 people consume groundwater exceeding 10 µg/L. Using simple previously published dose–response relationships, this is estimated to have given rise to 700 (prevalence) cases of skin cancer and around 10 cases of premature avoidable mortality/annum from internal (lung, liver, bladder) cancers—that latter value is on the order of just 0.001% of internal cancers in Gujarat, reflecting the relative low groundwater arsenic hazard in Gujarat State.

**Electronic supplementary material:**

The online version of this article (10.1007/s10653-020-00655-7) contains supplementary material, which is available to authorized users.

## Introduction

Arsenic (As) is a toxic element, found in more than 200 minerals in nature (Thornton and Farago 1997; Ravenscroft et al. 2009) with arsenic being released into groundwater under specific biogeochemical and hydrogeological conditions (Islam et al. 2004; Guo et al. 2011). In many parts of the world, arsenic-contaminated groundwater is used for drinking water and irrigation (Nickson et al. 2005; Rahman and Hasegawa 2011). The long-term consumption of arsenic may greatly increase the risk of skin cancers, bladder cancers, lung cancers, cardiovascular disease and other detrimental health outcomes (Chen and Ahsan 2004; Chowdhury et al. 2000). The provisional guideline value of arsenic in drinking water established by the World Health Organization (WHO) is 10 μg/L (WHO/UNICEF 2018); however, an increasing number of studies have pointed to detrimental health outcomes for exposure at lower arsenic concentrations (Medrano et al. 2010; García-Esquinas et al. 2013; Monrad et al. 2017; Moon et al. 2017; Polya et al. 2019b; Ahmad et al. 2020).

Groundwater arsenic contamination (WHO/UNICEF 2018; Bhattacharya et al. 2017; Bretzler and Johnson 2015) is the most substantive contributor to preventable detrimental health outcomes arising from chemicals such as F, Mn, Pb, pesticides in drinking water (Smith et al. 2000). As many as 100,000 preventable deaths may arise each year from exposure to such groundwater arsenic across the globe (Polya et al. 2019a, b; Smith et al. 2000), particularly in densely populated (van Geen 2008) areas in south and south-east Asia (Polya and Charlet 2009; Fendorf et al. 2010), Bangladesh (Argos et al. 2010, Flanagan et al. 2012), Pakistan (Podgorski et al. 2017) and India (Chakraborti et al. 2004).

Although there have been numerous studies of arsenic-contaminated groundwaters utilized for domestic consumption (e.g. Chatterjee et al. 1995; Chowdhury et al. 1999, 2000; Chakraborti et al. 2003), relatively few studies have produced hazard prediction maps indicating the spatial distribution of groundwater arsenic for whole districts or states in India (Buragohain and Sarma 2012; Ghosh et al. 2004, 2019). However, such maps have been generated for other regions (Amini et al. 2008; Winkel et al. 2008) and countries (Lado et al. 2008; Sovann and Polya 2014; Bretzler et al. 2017), notably including Bangladesh (Kinniburgh and Smedley 2001) and Pakistan (Podgorski et al. 2017).

Spatial geostatistical models used to predict the distribution of groundwater contaminants include logistic regression (Winkel et al. 2008; Ayotte et al. 2017; Podgorski et al. 2017; Bretzler et al. 2017) Tyson polygons (Ghosh et al. 2019), ordinary Kriging (Ghosh et al. 2019; Sovann and Polya 2014), regression Kriging (Sovann and Polya 2014), and random forest models (Podgorski et al. 2018). Methods such as logistic regression and random forest find statistical relationships between a target variable and predictor variables in order to make predictions (Winkel et al. 2008; Ayotte et al. 2017; Podgorski et al. 2017; Bretzler et al. 2017; Podgorski et al. 2018). Such methods can be used to consider a variety of environmental factors that may act as proxies or have a direct relationship to the release and accumulation of arsenic in groundwaters. Due to the often highly heterogeneous distribution of groundwater arsenic in sedimentary aquifers, modelling based on a binary target variable to produce probabilities, such as logistic regression, is often performed rather than attempting to predict a continuous variable.

As a preliminary step to developing a comprehensive model of the spatial distribution of arsenic in groundwaters across India, we (1) present logistic regression-based geostatistical models of the distribution of arsenic in groundwaters in the state of Gujarat, (2) outline methods permitting model results to be rendered as a pseudo-contour map of likely concentrations and (3) combine the modelled arsenic hazard with simple exposure route and dose–response models to provide plausible estimates of detrimental health outcomes in Gujarat that can be attributed to arsenic in drinking water.

## Materials and methods

### Study area

**Fig. 1:**
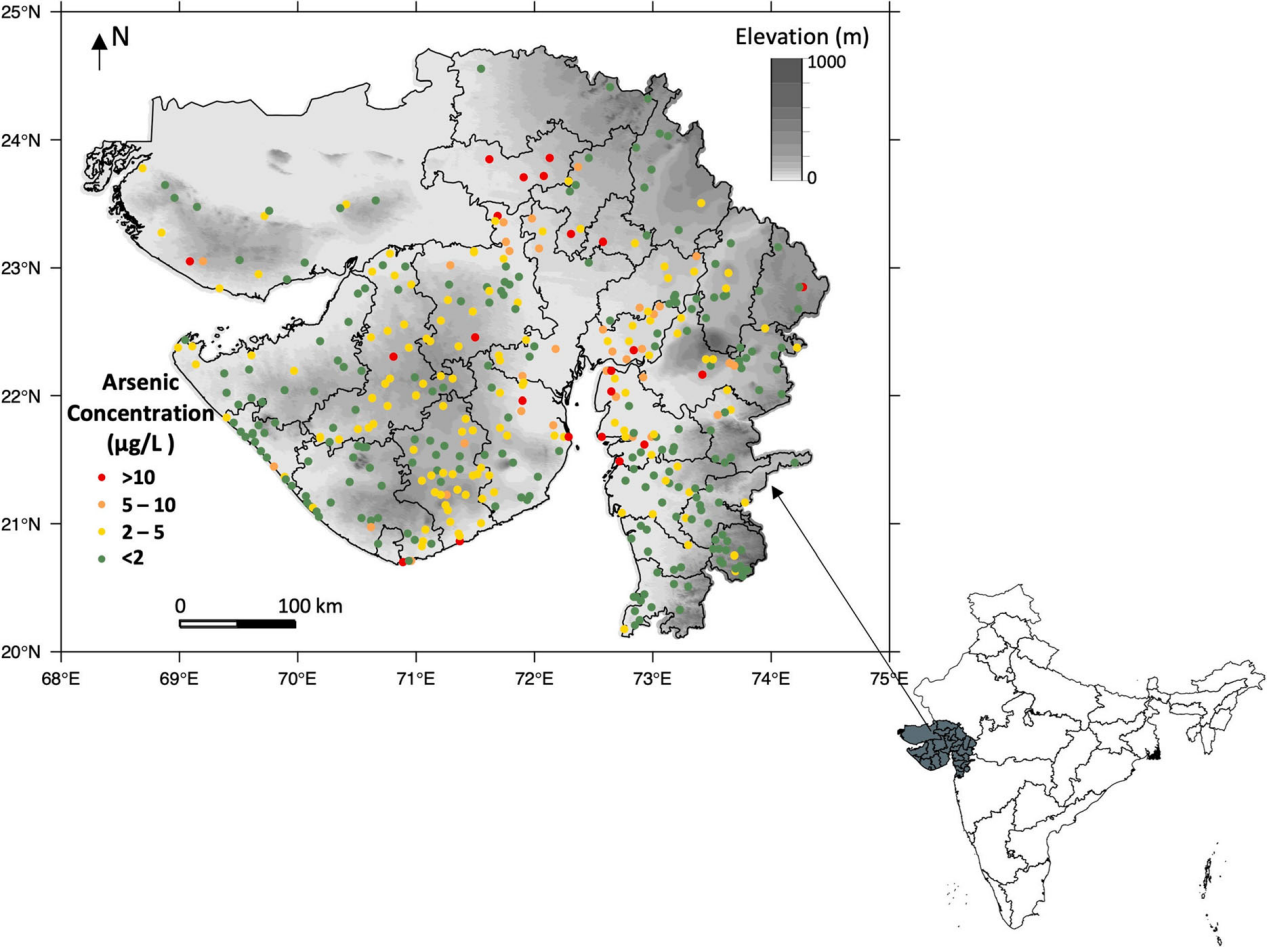
The location of Gujarat State and distribution of groundwater arsenic concentrations used in modelling

Gujarat is located between 20° 06′ and 24° 42′ north latitude and 68° 10′ to 74° 28′ east longitude, with an area of 196,024 sq. km (CGWB 2016) (Fig. 1). The population of Gujarat State is 70,445,000 (Chandramouli 2011). Gujarat has nearly 1600 km of coastline which is the longest coastline in India (CGWB 2016). Diverse climatic, topographic and geological and physiographic conditions result in diversification of groundwater conditions in different parts of Gujarat State (Sharma and Kumar 2008).

### Dataset compilation

Groundwater arsenic data from throughout Gujarat were obtained from surveys conducted by the Central Ground Water Board of India (CGWB) in 2015 (CGWB 2016). The CGWB collected groundwater samples from dug wells, tube wells and bore wells during May 2015, which is the end of dry season shortly before the onset of monsoon and analysed for arsenic by a colorimetric method using a visible spectrophotometer with an implied detection limit of around 1 μg/L. Of the 599 samples reported, (1) 183 samples for which the arsenic concentration was recorded as “nd” we have taken to have not been analysed and have excluded from the dataset; (2) a further 18 samples for which arsenic concentrations were reported without location data were also excluded from the dataset, leaving 398 datapoints with both groundwater arsenic and location data (Fig. 1): of these only 6% showed arsenic concentrations greater than 10 μg/L, with the maximum reported arsenic concentration being 26 μg/L. The frequency distribution of groundwater arsenic concentrations is shown in Fig. S1.

Potential independent variables (*n* = 28) related to geology, hydrology, soil properties, climate, and topography were compiled from a variety of sources, many based on or relying upon remote sensing (Table S1). These variables were initially chosen based on established and proposed relationships with the release and enrichment of groundwater arsenic (Smedley and Kinniburgh 2002; Islam et al. 2004; McArthur et al. 2004; Charlet and Polya 2006; Polya and Charlet 2009; Rodríguez-Lado et al. 2013; Polya and Middleton 2017; Podgorski et al. 2018; Polya et al. 2019a, b) and prepared to predict the distribution of groundwater arsenic in Gujarat State. The resolution and sources (Trabucco and Zomer 2009, 2010; ISRIC 2017; Hijmans et al. 2005; Hengl 2018; Fan et al. 2013; The World Bank 2017; Pelletier et al. 2016; Hartmann and Moosdorf 2012) of the independent variables dataset are shown in Table S1.

### Dataset preparation

The six thresholds of 10 μg/L, 5 μg/L, 4 μg/L, 3 μg/L, 2 μg/L and 1 μg/L were used to create binary datasets for creating six different geostatistical models. These values were chosen based on being the WHO provisional guideline of 10 μg/L and due to 85% of arsenic concentrations in the dataset being in the range of 1 to 5 μg/L. Of the 398 groundwater arsenic concentrations, 24 (6%), 57 (14%), 78 (20%), 124(31%), 185 (46%) and 301 (76%) arsenic concentrations exceeded 10 μg/L, 5 μg/L, 4 μg/L, 3 μg/L, 2 μg/L and 1 μg/L, respectively. The dataset was converted into high and low classes by assigning one to all arsenic concentrations > threshold concentrations and zero to all arsenic concentrations ≤ the threshold concentrations. The converted dataset was randomly divided into training (80%) and testing (20%) datasets maintaining the same ratio of low to high values as in the entire dataset.

### Statistical modelling

In this study, we used the logistic regression models to predict arsenic contamination in Gujarat groundwaters. Logistic regression uses a logistic function to predict a binary dependent variable with the probability between 0 and 1 (Hosmer et al. 2013). In this case, the binary dependent variable represents whether or not groundwater arsenic concentration exceeds a given threshold. The logistic function is as follows:

$$\log \left( {\frac{{P\left( {y = 1} \right)}}{{1 - P\left( {y = 1} \right)}}} \right) = \log \left( {\frac{{P\left( {y = 1} \right)}}{{ P\left( {y = 0} \right)}}} \right) = \beta_{0} + \beta_{1} x_{1} + \cdots + \beta_{n} x_{n}$$

$$\frac{{P\left( {y = 1} \right)}}{{1 - P\left( {y = 1} \right)}} = {\text{odds}} = \exp \left( {\beta_{0} + \beta_{1} x_{1} + \cdots + \beta_{n} x_{n} } \right)$$

$$P\left( {y = 1} \right) = \frac{1}{{1 + \exp \left( { - \left( {\beta_{0} + \beta_{1} x_{1} + \cdots + \beta_{n} x_{n} } \right)} \right)}}$$

where $P\left( {y = 1} \right)$ and $P\left( {y = 0} \right)$ are the probability of the dependent variable being 1 or 0; $x_{1} \ldots x_{n}$ are the independent variables; $\beta_{0} \ldots \beta_{n}$ are the regression intercept and other coefficients.

Multicollinearity is a statistical phenomenon in which predictor variables of a logistic regression model are highly correlated. The existence of collinearity increases the variances of parameter estimates and thus leads to erroneous inferences about the relationship between dependent and independent variables (Midi et al. 2010). Variance inflation factor (VIF) quantifies the severity of multicollinearity of independent variables (predictors) in regression analysis (Franke 2010). It was used for independent variable selection in this study.

$${\text{VIF}} = \frac{1}{{1 - R^{2} }}$$

where $R^{2}$ is the coefficient of determination, $R^{2} = 1 - {\text{e}}^{{ - \frac{D}{n}}}$ (*D* is the test statistic of the likelihood ratio test, *n* is the sample size.)

The empirical judgment method is that if VIF > 10 then multicollinearity is high (Franke 2010).

We used stepwise variable selection in which Akaike information criterion (AIC) is used as criterion for removing or adding variables to determine final logistic regression models. AIC is an estimator of the complexity and goodness of fit of statistical models (Akaike 1974).

$${\text{AIC}} = 2k - 2 \ln \left( L \right)$$

where $k$ is the number of parameters; $L$ is the Likelihood of the model.

The objectively preferred variable combination in stepwise selection was the one with the lowest AIC value, providing the best combination of performance and complexity.

### Variable selection

Based on their known or potential relationships to arsenic occurrence in groundwater, twenty-eight independent variables (see Table S1), including twenty-four continuous variables and four categorical variables, were considered for potential use in logistic regression modelling. In order to help identify effective independent variables, univariate logistic regressions were run for each of six thresholds on the training dataset which is consistent with the dataset used for logistic regression analysis. The significance of each independent variable was assessed through its *p* value tested by the analysis of variance (AVOVA) type II test (Pearce and Ferrier 2000). Independent variables with *p* values < 0.05 (within the 95% confidence interval) were retained for further selection. Multicollinearity of the continuous variables following the univariate analysis was then calculated on the training dataset at each threshold. Predictor variables with a variance inflation factor (VIF) > 10 were removed on the basis of strong multicollinearity. The univariate regression and multicollinearity analysis were repeated 1000 times in order to avoid the random bias produced by specific splitting of training and testing datasets at one time. The averaged *p* value and VIF were used to determine the addition or removal of variables during variable selection.

### Logistic regression analysis

Logistic regression analysis was run on the training dataset for each of six thresholds using a stepwise selection of variables (both directions), which removes or adds variables according to their improvement to the Akaike information criterion (AIC). The Hosmer–Lemeshow goodness-of-fit test (Hosmer et al. 2013) was also used on the testing dataset to determine the accuracy of regressions at the 95% confidence level, such that there is no significant difference between the fitted values and observed values if the *p* value is > 0.05. In order to avoid introducing bias to the model by performing only a single split of training and testing datasets, logistic regressions were performed 1000 times with the Hosmer–Lemeshow goodness-of-fit test. The logistic regression models passing the Hosmer–Lemeshow goodness-of-fit test (*p* value is > 0.05) provided various variable combinations determined by AIC values. The different combinations of variables of the logistic regressions passing the Hosmer–Lemeshow goodness-of-fit test were counted. The mean of coefficients of each combination passing the Hosmer–Lemeshow goodness-of-fit test were utilized as the coefficients of the model.

The true-positive rate (sensitivity) and true-negative rate (specificity) were calculated on both the entire dataset and testing datasets passing the Hosmer–Lemeshow goodness-of-fit test for each of the six thresholds. Plotting sensitivity against specificity for the range of probability cut-off values from 0 to 1 on the entire dataset produced a receiver operating characteristic (ROC) curve and the associated area under the ROC curve (AUC), which generally ranges from 0.5 (no predictive capability) to 1 (perfect predictive capability) (Fawcett 2006). Mean AUC values were also calculated on the test dataset of each logistic regression. The largest AUC value among the variable combinations passing the Hosmer–Lemeshow goodness-of-fit test was used to select the final model.

### Hazard and potential exposure maps

The final logistic regression models were utilized to calculate the probability of groundwater arsenic concentration exceeding each of the threshold concentrations. The sensitivity, accuracy, and specificity of the final models were plotted against cut-offs. The cut-off values at which sensitivity and specificity are equal were used to classify whether arsenic concentrations exceed the given thresholds (Podgorski et al. 2017). These were then used to generate a pseudo-contour map of groundwater arsenic concentrations, which was combined with population density (Pages et al. 2018) to generate a potential exposure map.

### Health risk estimation

Based on the potential exposure map, we used dose response functions for arsenic-induced cancers to evaluate the health effects of exposure to groundwater arsenic in Gujarat.

1. Prevalence ratio of arsenic-induced skin cancer as a function of arsenic concentration, *c*, and age, *t* (Brown et al. 1989).

$$p\left( {c,t} \right) = 1 - \exp \left( { - \left( {q_{1} c + q_{2} c^{2} } \right)\left( {t - m} \right)^{k} H\left( {t - m} \right)} \right)$$

where $p\left( {c, t} \right)$ denotes prevalence ratio of the gender with arsenic-induced skin cancer; $c$ denotes arsenic concentration, μg/L; $t$ denotes age, year; $q_{1} ,q_{2} , k, m$ are the nonnegative parameters, listed in Table S2;

*H*(*t* − *m*) denotes the Heaviside function with $H\left( {t - m} \right) = 0$ for $t < m$ and $H\left( {t - m} \right) = 1$ for $t \ge m$.

(2) Incidence rate of arsenic-induced internal cancer (lung cancer, bladder cancer, liver cancer) as a function of arsenic concentration, c, and age, t (NRC 1999, 2001; Yu et al. 2003).

$$h\left( {c,t} \right) = k\left( {q_{1} c + q_{2} c^{2} } \right)\left( {t - m} \right)^{k - 1} H\left( {t - m} \right)$$

where $h\left( {c, t} \right)$ denotes incidence rate of the gender with arsenic-induced internal cancer, per year; $c$ denotes arsenic concentration, μg/L; $t$ denotes age, year; $q_{1} ,q_{2} , k, m$ are the nonnegative parameters, listed in Table S2;

*H*(*t* − *m*) denotes the Heaviside function with $H\left( {t - m} \right) = 0$ for $t < m$ and $H\left( {t - m} \right) = 1$ for $t \ge m$.

## Results and discussion

### Logistic regression models

The univariate regression and multicollinearity analysis retained 10, 15, 16, 13, 9 and 2 independent variables for models with thresholds of 10 μg/L, 5 μg/L, 4 μg/L, 3 μg/L, 2 μg/L, and 1 μg/L, respectively (Table 1). Of the 1000 logistic regression iterations performed, 707, 535, 679, 736, 858 and 473 regression runs passed the Hosmer–Lemeshow goodness-of-fit test of models using the thresholds of 10 μg/L, 5 μg/L, 4 μg/L, 3 μg/L, 2 μg/L, and 1 μg/L, respectively. The variables appearing in the regressions passing the Hosmer–Lemeshow goodness-of-fit test for each of six thresholds are listed in Table S3.

**Table 1:** Univariate logistic regressions and multicollinearity analysis for models with 1–10 μg/L as thresholds for groundwater arsenic

Variables	Thresholds											
	10 μg/L		5 μg/L		4 μg/L		3 μg/L		2 μg/L		1 μg/L	
	*p* value	VIF	*p* value	VIF	*p* value	VIF	*p* value	VIF	*p* value	VIF	*p* value	VIF
Actual evapotranspiration	3.80 × 10^−1^		4.69 × 10^−1^		**1.38 **× **10**^**−2**^	103	1.81 × 10^−3^	94.11	**3.34 **× **10**^**−4**^	81.8	6.94 × 10^−1^	
Aridity	**2.35 **× **10**^**−2**^	**2.43**	**4.11 **× **10**^**−3**^	**2.91**	**9.97 **× **10**^**−6**^	12	**1.95 **× **10**^**−7**^	**7.13**	**1.43 **× **10**^**−7**^	**7.36**	7.45 × 10^−1^	
Calcisols	1.78 × 10^−1^		1.10 × 10^−1^		**3.78 **× **10**^**−2**^	**2.12**	1.76 × 10^−1^		3.96 × 10^−1^		1.61 × 10^−1^	
Fluvisols	6.85 × 10^−2^		**1.43 **× **10**^**−3**^	**1.26**	**1.62 **× **10**^**−4**^	**1.54**	**1.86 **× **10**^**−3**^	**1.56**	1.02 × 10^−1^		5.04 × 10^−1^	
Gleysols	5.10 × 10^−1^		1.35 × 10^−1^		2.97 × 10^−1^		6.73 × 10^−1^		7.26 × 10^−1^		7.36 × 10^−1^	
Potential evapotranspiration	**6.71 **× **10**^**−4**^	**2.87**	**2.57 **× **10**^**−6**^	**2.95**	**3.49 **× **10**^**−6**^	**4.66**	**5.98 **× **10**^**−8**^	**3.89**	**6.54 **× **10**^**−8**^	**4.96**	**2.19 **× **10**^**−2**^	**1.36**
Precipitation	2.61 × 10^−1^		3.13 × 10^−1^		**6.88 **× **10**^**−3**^	87.94	**5.72 **× **10**^**−4**^	71.56	**1.43 **× **10**^**−4**^	54.75	5.99 × 10^−1^	
Priestly–Taylor alpha coefficients	8.57 × 10^−2^		5.24 × 10^−2^		**1.02 **× **10**^**−3**^	40.22	**1.56 **× **10**^**−5**^	33.96	**1.62 **× **10**^**−6**^	33.92	1.48 × 10^−1^	
Slope	**4.79 **× **10**^**−5**^	**1.69**	**1.99 **× **10**^**−5**^	**2.04**	**9.14 **× **10**^**−6**^	**1.91**	**5.86 **× **10**^**−6**^	**1.90**	**5.56 **× **10**^**−4**^	**2.16**	1.02 × 10^−1^	
Soil and sedimentary deposit thickness	**3.00 **× **10**^**−4**^	**1.66**	**1.00 **× **10**^**−6**^	**2.07**	**2.10 **× **10**^**−6**^	**2.41**	**3.74 **× **10**^**−4**^	**2.11**	**4.11 **× **10**^**−2**^	**1.88**	5.15 × 10^−1^	
Soil cation exchange capacity	6.95 × 10^−2^		**7.79 **× **10**^**−3**^	**4.13**	**3.12 **× **10**^**−2**^	**4.75**	3.08 × 10^−1^		4.82 × 10^−1^		1.22 × 10^−1^	
Soil organic carbon content	6.50 × 10^−1^		1.90 × 10^−1^		2.76 × 10^−1^		7.43 × 10^−1^		7.38 × 10^−1^		1.66 × 10^−1^	
Soil organic carbon density	5.91 × 10^−1^		6.27 × 10^−1^		3.78 × 10^−1^		5.97 × 10^−1^		5.52 × 10^−1^		6.49 × 10^−1^	
Soil organic carbon stock	5.65 × 10^−1^		9.22 × 10^−2^		7.15 × 10^−2^		5.59 × 10^−1^		4.57 × 10^−1^		2.03 × 10^−1^	
Soil pH	1.11 × 10^−1^		1.88 × 10^−1^		**3.54 **× **10**^**−2**^	**2.46**	**7.03 **× **10**^**−3**^	**2.36**	**8.03 **× **10**^**−4**^	**2.69**	2.79 × 10^−1^	
Soil water capacity	**1.89 **× **10**^**−2**^	**1.34**	**2.27 **× **10**^**−3**^	**5.28**	**1.61 **× **10**^**−2**^	**6.36**	1.56 × 10^−1^		6.95 × 10^−1^		8.73 × 10^−2^	
Solonchaks	5.01 × 10^−1^		5.56 × 10^−1^		7.75 × 10^−1^		4.30 × 10^−1^		**4.68 **× **10**^**−2**^	**2.35**	6.09 × 10^−1^	
Temperature	**2.98 **× **10**^**−3**^	**3.50**	**1.35 **× **10**^**−5**^	**3.40**	**5.42 **× **10**^**−4**^	**8.65**	**4.23 **× **10**^**−4**^	**7.01**	**2.85 **× **10**^**−2**^	**7.34**	**2.33 **× **10**^**−2**^	**1.36**
Topographic wetness index	**2.45 **× **10**^**−3**^	**2.29**	**1.19 **× **10**^**−5**^	**2.55**	**9.52 **× **10**^**−7**^	**2.46**	**4.20 **× **10**^**−7**^	**2.76**	**1.39 **× **10**^**−4**^	**3.10**	4.65 × 10^−1^	
Volume percentage of coarse fragments	1.92 × 10^−1^		**7.90 **× **10**^**−3**^	**1.25**	**2.48 **× **10**^**−3**^	**1.52**	**2.20 **× **10**^**−2**^	**1.49**	**4.51 **× **10**^**−2**^	**1.50**	1.96 × 10^−1^	
Water table depth	**1.50 **× **10**^**−2**^	**1.76**	**6.02 **× **10**^**−3**^	**1.76**	**3.15 **× **10**^**−3**^	**1.83**	**2.30 **× **10**^**−2**^	**1.90**	1.70 × 10^−1^		2.46 × 10^−1^	
Weight percentage of clay particles	7.88 × 10^−2^		**1.97 **× **10**^**−2**^	**4.55**	**3.11 **× **10**^**−2**^	**5.46**	2.41 × 10^−1^		7.11 × 10^−1^		1.49 × 10^−1^	
Weight percentage of sand particles	2.22 × 10^−1^		**2.77 **× **10**^**−2**^	**4.70**	**1.59 **× **10**^**−2**^	**5.75**	1.11 × 10^−1^		6.19 × 10^−1^		1.73 × 10^−1^	
Weight percentage of silt particles	5.51 × 10^−1^		6.63 × 10^−1^		2.67 × 10^−1^		2.01 × 10^−1^		4.49 × 10^−1^		7.06 × 10^−1^	
Acid plutonic rocks	4.10 × 10^−1^		1.95 × 10^−1^		1.23 × 10^−1^		**4.50 **× **10**^**−2**^		1.27 × 10^−1^		3.65 × 10^−1^	
Basic volcanic rocks	**3.03 **× **10**^**−2**^		**6.02 **× **10**^**−3**^		**1.39 **× **10**^**−2**^		**2.46 **× **10**^**−2**^		3.57 × 10^−1^		6.77 × 10^−1^	
Metamorphic rocks	5.67 × 10^−1^		5.37 × 10^−1^		3.06 × 10^−1^		2.75 × 10^−1^		3.30 × 10^−1^		3.38 × 10^−1^	
Sedimentary rocks	**2.89 **× **10**^**−2**^		**1.28 **× **10**^**−4**^		**2.24 **× **10**^**−3**^		**2.86 **× **10**^**−3**^		1.03 × 10^−1^		6.58 × 10^−1^	

Bold shows where *p* values are less than 0.05 and variance inflation factor (VIF) values are less than 10

The optimum combinations of independent variables in the final model for each threshold were determined using the areas under the ROC curve (AUC). Both the AUC calculated using entire dataset and testing datasets of regressions passing the Hosmer–Lemeshow goodness-of-fit test were very similar. Six variable combinations with highest AUC values (Table 2) were selected as final models. The coefficients and intercepts of normalized variables and their standard deviations in final models are summarized in Table 3. However, many other variable combinations not selected for various thresholds may also have good predictive capabilities, as evidenced by high AUC values, see Tables S4–S8.

**Table 2:** Variable combinations and AUC values of final models in 1000 logistic regressions using thresholds of 1–10 μg/L for groundwater arsenic

No.	Threshold (μg/L)	Variable combination	AUC value
1	10	Potential evapotranspiration, slope, soil water capacity	0.83
2	5	Soil and sedimentary deposit thickness, potential evapotranspiration, slope	0.79
3	4	Topographic wetness index, potential evapotranspiration, fluvisols	0.77
4	3	Aridity, fluvisols, temperature	0.76
5	2	Aridity, temperature	0.71
6	1	Evapotranspiration	0.60

**Table 3:** Intercepts, coefficients, and their standard deviations of normalized predictor variables of final models in 1000 logistic regression runs using thresholds of 1–10 μg/L for groundwater arsenic

Variables	Thresholds											
	10 μg/L		5 μg/L		4 μg/L		3 μg/L		2 μg/L		1 μg/L	
	Coefficient	Standard deviation	Coefficient	Standard deviation	Coefficient	Standard deviation	Coefficient	Standard deviation	Coefficient	Standard deviation	Coefficient	Standard deviation
Intercept	− 1.75	0.36	− 3.36	0.10	− 7.94	0.52	− 2.14	0.21	− 0.22	0.13	0.34	0.07
Aridity							− 6.99	0.72	− 5.99	0.49		
Fluvisols					2.58	2.79	1.97	0.11				
Potential evapotranspiration	3.47	0.39	2.67	0.22	2.76	0.33					1.68	0.15
Slope	− 25.19	3.6	− 10.51	0.21								
Soil and sedimentary deposit thickness			1.18	0.05								
Soil water capacity	− 3.55	0.27										
Temperature							2.94	0.2	1.95	0.23		
Topographic wetness index					4.83	0.58						

**Fig. 2:**
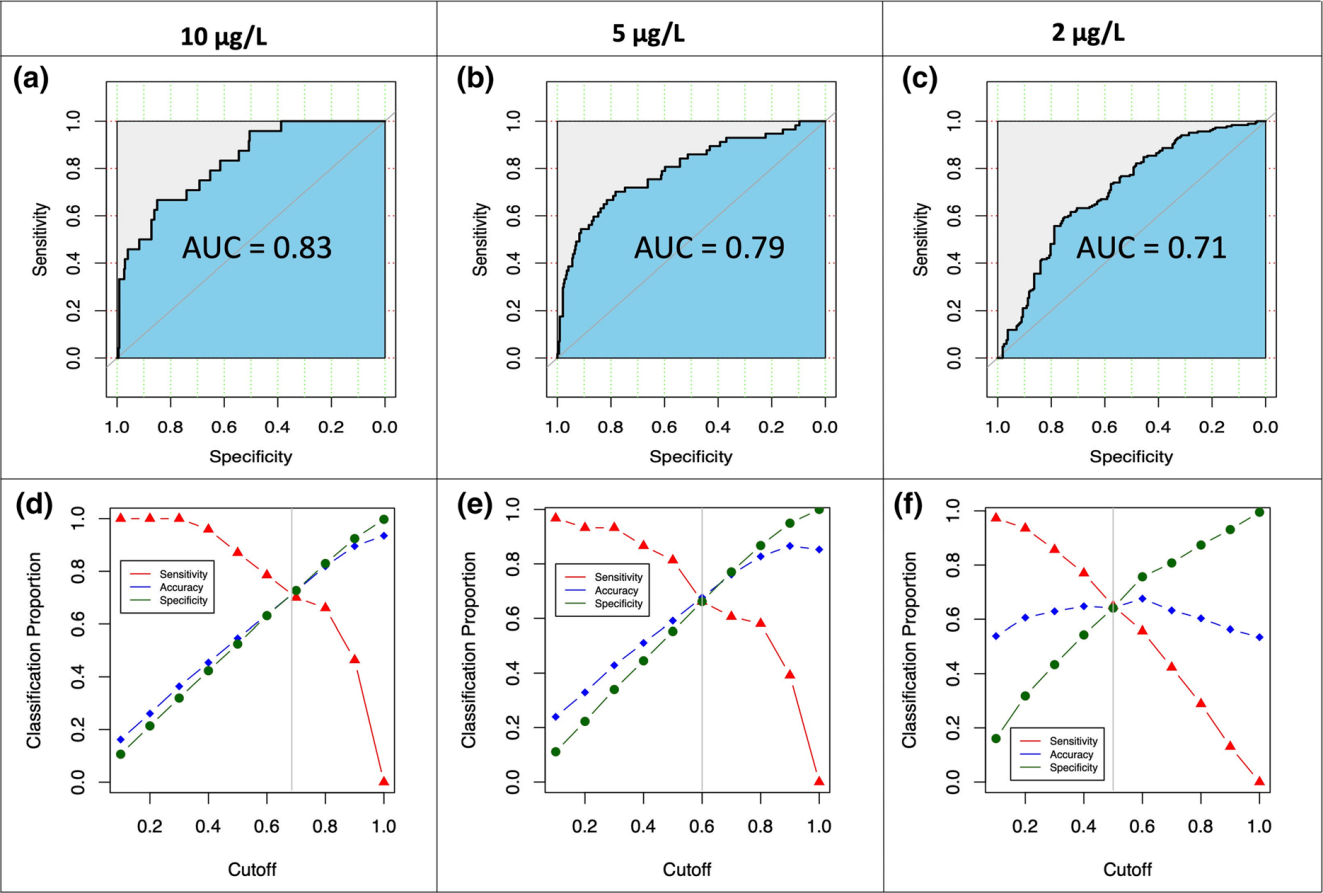
ROC curves of final logistic regression models with **a** 10 μg/L, **b** 5 μg/L, and **c** 2 μg/L as thresholds for groundwater arsenic in Gujarat State, India. Plots of sensitivity (true-positive rate), specificity (true-negative rate) and accuracy against cut-offs of the final logistic regression models with **d** 10 μg/L, **e** 5 μg/L, and **f** 2 μg/L as thresholds for CGWB (2016) dataset for groundwater arsenic in Gujarat State, India

The AUC values indicate that models with thresholds of 10 μg/L (Fig. 2), 5 μg/L (Fig. 2), 4 μg/L (Fig. S2), 3 μg/L(Fig. S2), and 2 μg/L (Fig. 2) perform well (AUC 0.71–0.83), whereas the classification performance of the 1 μg/L model is not satisfactory (AUC 0.60, Fig. S2), which may be due to detection limits of the arsenic analysis. The 1 μg/L model was therefore excluded from the further consideration. The crossover between sensitivity (true-positive rate) and specificity (true-negative rate) against cut-offs (Figs. 2 and S3) were utilized to determine high-risk areas of groundwater arsenic concentrations.

### Predictor variables

Eight predictor variables were included in the final models to predict the distribution of groundwater arsenic in Gujarat and can be grouped into three categories: (1) climate variables, (2) geological variables and (3) topographic variables (Fig. S5). Positive coefficients were found for fluvisols, soil and sedimentary deposit thickness, potential evapotranspiration, temperature, and topographic wetness index, whereas negative coefficients were found for aridity, slope, and soil water capacity.

Climate variables (temperature, potential evapotranspiration, and aridity) in final models relate to arsenic accumulation in aquifers significantly. High temperature promotes the evapotranspiration and can increase drought. The combination of high temperature, high evapotranspiration and low aridity index (average precipitation/potential evapotranspiration) can increase the evaporative concentration of groundwater and hence increase arsenic concentrations, particularly in inland and/or enclosed basins in arid or semi-arid climates (Smedley and Kinniburgh 2002; Ravenscroft et al. 2009; Alarcón-Herrera et al. 2013).

Fluvisols and soil and sedimentary deposit thickness are also conducive to the enrichment of arsenic in groundwaters. Fluvisols are genetically young soils in alluvial deposits (IUSS 2015). Previous studies (Ahmed et al. 2004; Chakraborti et al. 2013; McArthur et al. 2001) have shown that arsenic pollution occurs dominantly in the alluvial deposits of major rivers which flow south and east from the Himalayas and Tibetan plateau, where rivers flow through the highest mountains with the largest rainfall and generate the greatest sedimentary deposit worldwide. The widely accepted mechanism of arsenic release into groundwaters in alluvial aquifers is the microbially mediated dissimilatory reductive dissolution of arsenic-bearing Fe oxides (Fe oxyhydroxides, hydroxides, and oxides) (Islam et al. 2004; Berg et al. 2007). The abundance of relatively young reactive organic matter in sedimentary deposits is plausibly causally linked to the occurrence of high arsenic concentrations in groundwaters (Rowland et al. 2007, 2011; Mukherjee et al. 2019). Hence, increased fluvisols, soil and sedimentary deposit thickness promote arsenic accumulation in groundwaters.

Low slope can be regarded as a proxy for slow groundwater flow, which suppresses the flushing of arsenic from groundwater systems. The gentle slope facilitates the accumulation of abundant organic matter within floodplains and alluvial deposits, arsenic-bearing Fe-oxyhydroxide minerals, and finer sediments (Shamsudduha and Uddin 2007; Shamsudduha et al. 2009). Then, arsenic is released into groundwaters by microbial activities, resulting in groundwater arsenic occuring in flat, low-lying areas where groundwater flows are sluggish; such areas include low-lying deltaic and floodplain areas (Shamsudduha et al. 2009).

### Hazard maps

**Fig. 3:**
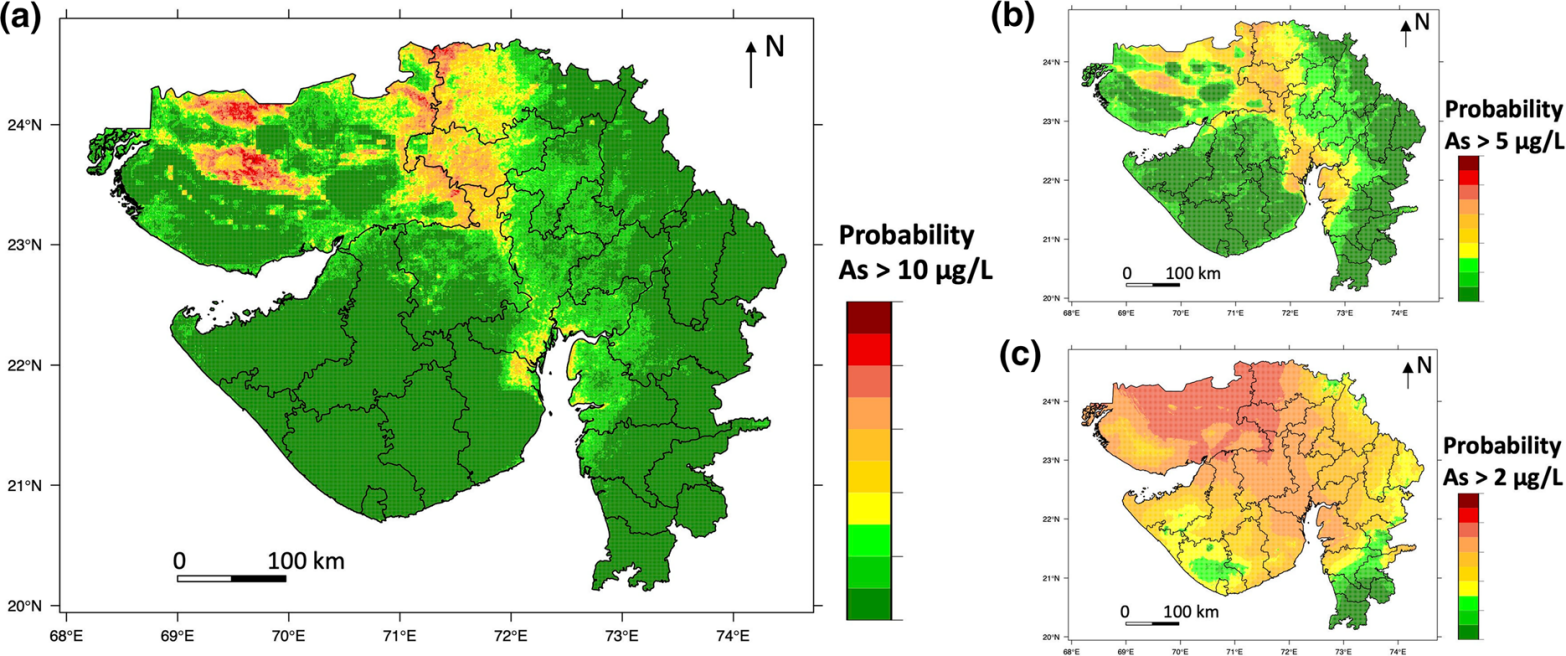
Hazard maps showing the probability of the geospatially modelled occurrences of groundwater arsenic concentration exceeding thresholds of **a** 10 μg/L, **b** 5 μg/L, and **c** 2 μg/L in Gujarat State, India

**Fig. 4:**
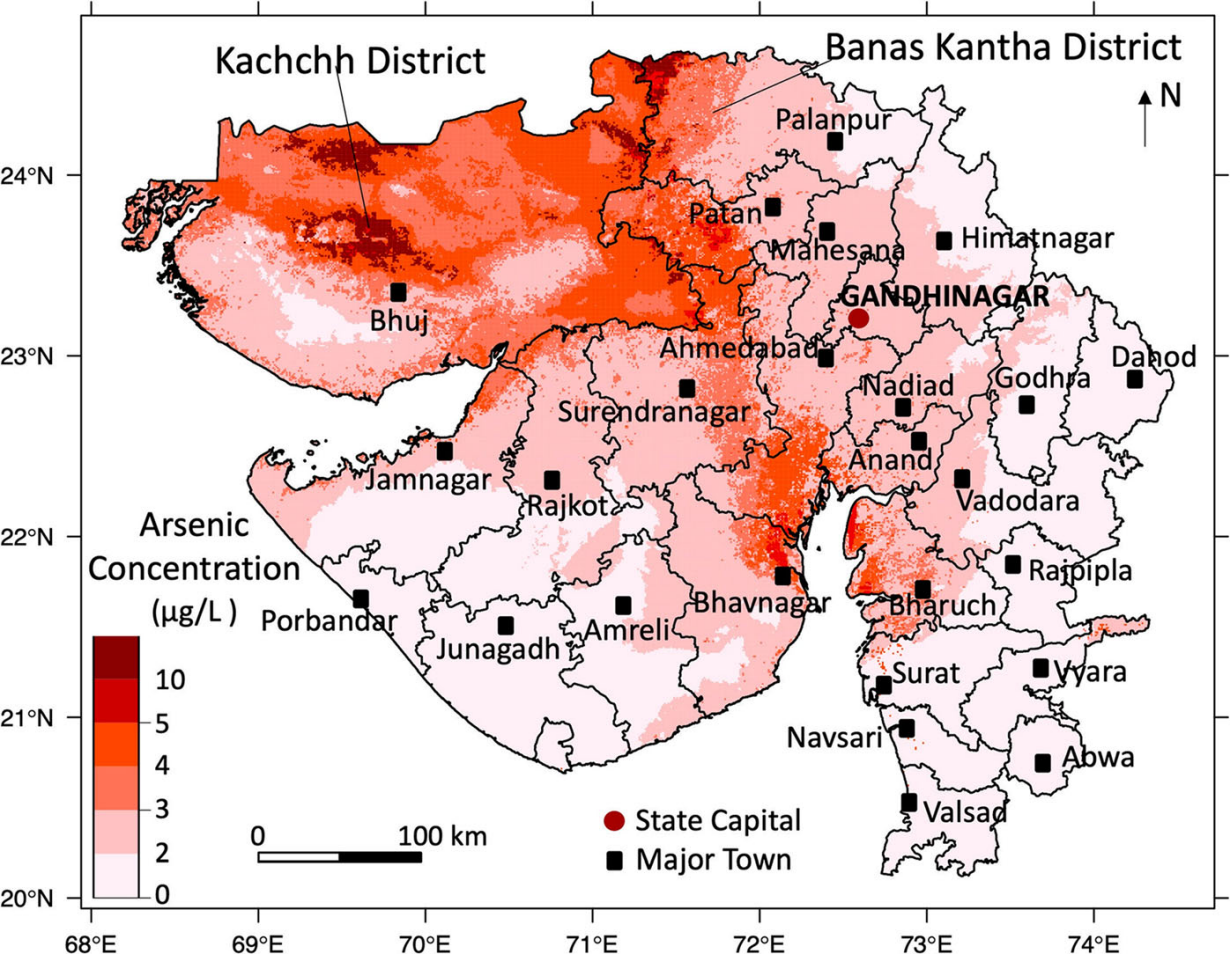
Pseudo-contour map of geospatially modelled groundwater arsenic hazard distribution in Gujarat. Contour boundaries surround the regions in which the modelled probability of groundwater arsenic exceeding the contour value is equal to the cut-off value for that concentration (being 0.5 for As = 2 µg/L; 0.57 for As = 3 µg/L; 0.61 for As = 4 µg/L; 0.66 for As = 5 µg/L; 0.69 for As = 10 µg/L)

The probabilities of arsenic concentration exceeding 10 μg/L, 5 μg/L, 4 μg/L, 3 μg/L and 2 μg/L were calculated by the 5 final models, and the probability maps of arsenic concentrations are shown in Figs. 3 and S4. The cut-offs where sensitivity equals specificity in the respective 5 final models (shown in Figs. 2 and S3) were 0.69 (10 μg/L), 0.66 (5 μg/L), 0.61 (4 μg/L), 0.57 (3 μg/L) and 0.50 (2 μg/L), which were used to create maps of the occurrence of arsenic concentration exceeding each of the thresholds. Figure 4 contains the pseudo-contour map of various concentrations of groundwater arsenic, combined from the individual hazard map of each of the thresholds. Of the 26 districts of Gujarat State as defined by the 2011 Indian Census (Chandramouli 2011), our map predicts that groundwater arsenic exceeds 10 μg/L in the northwest, northeast and south-east parts of Kachchh district and the north-western and south-western part of Banas Kantha district. In comparison, a pseudo-contour map of groundwater arsenic determined using a fixed cut-off of 0.50 indicates more widely varying higher concentrations (Fig. S6).

The pseudo-contour map of arsenic concentrations (Fig. 4) shows a similar spatial pattern to the distribution map of soil organic carbon content (Fig. S7), which is not one of the predictor variables. Dissolved organic matter is the main driver of microbe-mediated reductive dissolution of arsenic-bearing Fe-oxyhydroxide (Fendorf et al. 2010). Other processes, including complexation of arsenic by dissolved humic substances, competitive sorption and electron shuttling reactions mediated by humic substances may also influence arsenic mobility in groundwaters (Guo et al. 2011; Mladenov et al. 2015). The amount and availability of organic carbon in sediments and soil affect the spatial variability of groundwater arsenic concentrations (McArthur et al. 2004; McArthur et al. 2011).

### Potential exposure map

**Fig. 5:**
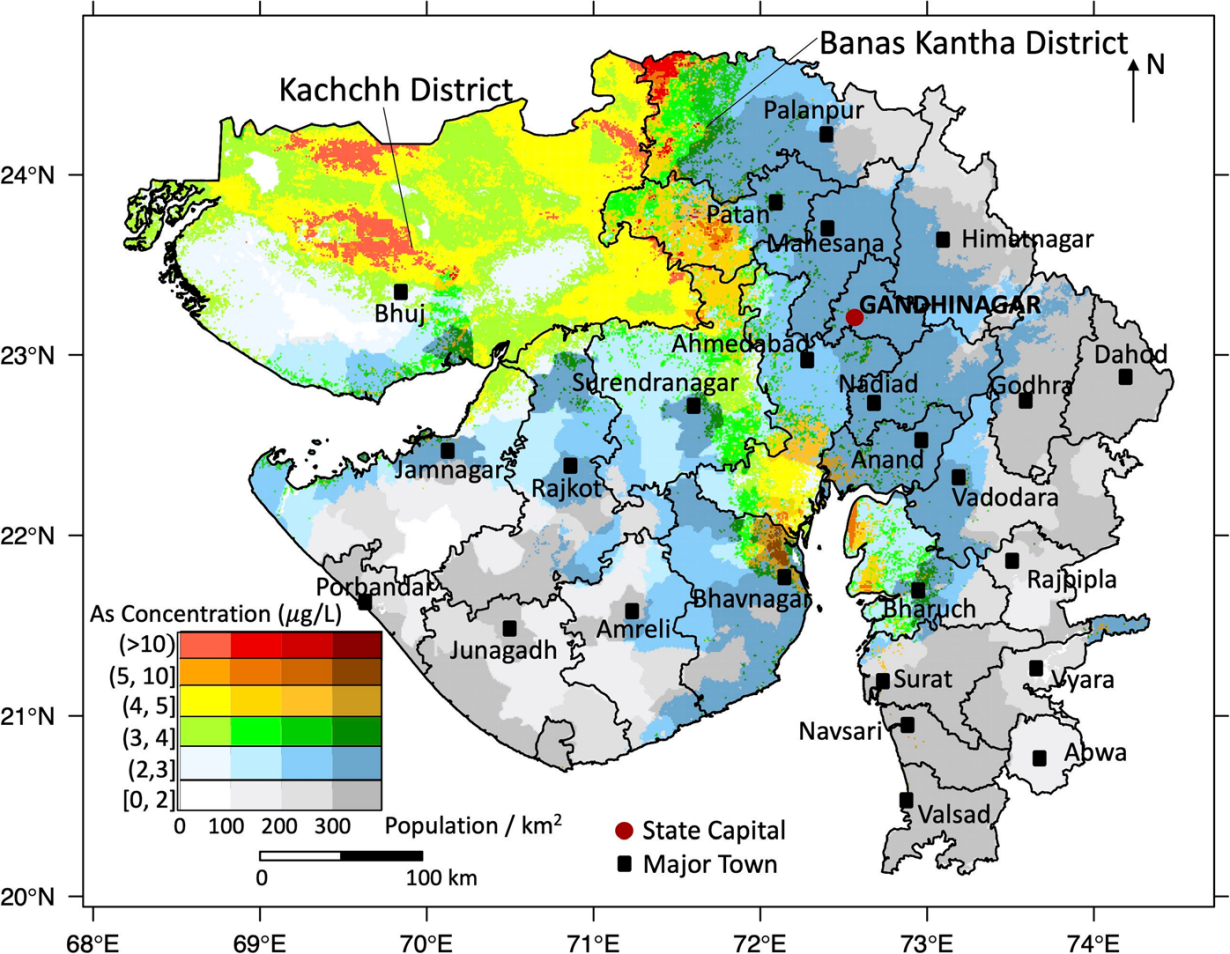
Population density (persons per 1 km^2^) co-plotted with modelled groundwater arsenic concentrations in Gujarat State, India. Cities are shown for illustrative purposes only. The proportion of people utilizing untreated groundwater for drinking purpose differs substantially between urban and rural areas, so this map should not be utilized as an exposure map without appropriate correction for groundwater usage

We combined the pseudo-contour map of varying arsenic concentrations with projected 2020 population density (Pages et al. 2018) to produce a potential exposure map showing the population living in areas with different groundwater arsenic concentrations (Fig. 5). Of a projected total population of Gujarat of 70,445,000 (Chandramouli 2011), approximately 122,000 (i.e. about 0.17% of total Gujarat population) live in areas where groundwater arsenic concentrations exceed 10 μg/L. The number of people living in areas with other groundwater arsenic concentrations is summarized in Table 4. In Gujarat State, only a low percentage of people (0.07%) were exposed to high arsenic groundwaters, and most people are likely to be exposed to low groundwater concentrations of arsenic. However, many studies (Medrano et al. 2010; Moon et al. 2017; Polya et al. 2019b; Ahmad et al. 2020) pointed out that low concentrations of arsenic also pose health risks to humans, although the harm is not as serious as that arising from higher arsenic concentrations in drinking water.

**Table 4:** Estimated population exposed to various groundwater arsenic concentration in Gujarat

Arsenic concentrations (μg/L)	Population living in areas with indicated range of groundwater arsenic concentrations	Population exposed to indicated range of groundwater arsenic concentrations
> 10	122,000	49,000
(5, 10)	206,000	82,000
(4, 5)	1,773,000	708,000
(3, 4)	5,011,000	2,000,000
(2, 3)	32,981,000	13,162,000
(0, 2)	30,351,000	12,113,000
Total	70,444,000	28,114,000

Accounting for 48% of rural household water supplied being through hand pumps and tube wells (The World Bank 2006) and 29% of urban households using untreated taps, bore wells, hand pumps and wells as water supply infrastructure (IIHS 2014), we estimate that approximately 49,000 people in Gujarat are exposed to elevated arsenic contamination (> 10 μg/L) through domestic consumption of groundwater. The population exposed to other arsenic concentrations in groundwaters is summarized in Table 4.

Health effects of exposure to groundwater arsenic in Gujarat (Table 5) estimated using dose response functions of arsenic-induced cancers (Brown et al. 1989; NRC 1999, 2001; Yu et al. 2003) include a prevalence of 670 cases of skin cancer arising from exposure to groundwater arsenic in Gujarat. However, in Gujarat, groundwater arsenic does not significantly contribute to internal cancers (lung cancer, bladder cancer, liver cancer) with a combined modelled incidence of only 12 cases—corresponding to just 0.001% of cancer-related fatalities in Gujarat. The low number of cancer cases modelled to be caused by groundwater arsenic reflects the relative low groundwater arsenic hazard in Gujarat State. These results are similar to those estimated by Yu et al. (2003) for low groundwater arsenic areas in Bangladesh (viz. Brahmaputra FP (Chandina regions), the Chittagong Coast (sandstone/shale regions), and the Terraces West/East (clays and alluvium regions) where mean groundwater arsenic concentrations are in the range of 1–6 μg/L. Notwithstanding this, there are method model and parameter uncertainties in the dose–response relations used and these warrant further investigation in order to obtain more accurate estimates of arsenic attributable health outcomes.

**Table 5:** Modelled health effects in exposure to groundwater arsenic hazard in Gujarat: prevalence of skin cancer and incidence of internal cancers (lung cancer, bladder cancer, liver cancer) for males and females in rural and urban areas

	Rural areas		Urban areas		
	Males	Females	Males	Females	Total
Prevalence of skin cancer (number of persons)	380	80	180	30	670
Incidence of internal cancers (fatalities per year)					
Lung cancer	8	0	4	0	12
Bladder cancer	0	0	0	0	0
Liver cancer	0	0	0	0	0

### Implications

The groundwater arsenic hazard and potential exposure maps for Gujarat produced in our study facilitate the calculation of the spatial distribution of groundwater arsenic attributable health outcomes. The predictive maps generated in this paper have high resolution and so provide a means of interpolating existing data (CGWB 2016), thereby providing value added to such existing datasets. Our arsenic distribution map, potential exposure map and associated health risk estimation of population present an obvious improvement in the rendering of both the detailed distribution of different arsenic concentrations in groundwaters and in estimates of the number of people potentially affected in Gujarat State. Our models also provide a basis for applying to other parts of India and globally, particularly useful for estimating populations at risk of exposure to different levels of hazard. Notwithstanding the utility of these models, we note that they are not intended to be an authoritative indicator of the quality of individual groundwater sourced tube-well water. Accordingly, these findings should be used with caution. In particular, the well-known significant local scale spatial heterogeneity in arsenic in groundwater indicates that wells should be tested individually in order to obtain the most robust assessment of groundwater arsenic hazard.

## Electronic supplementary material

Below is the link to the electronic supplementary material.

Supplementary material 1 (DOCX 45 kb)

Supplementary material 2 (TIFF 530 kb)

Supplementary material 3 (TIFF 1636 kb)

Supplementary material 4 (TIFF 1483 kb)

Supplementary material 5 (TIFF 9625 kb)

Supplementary material 6 (TIFF 2926 kb)

Supplementary material 7 (TIFF 1587 kb)

Supplementary material 8 (TIFF 1440 kb)
