# Supplementary material for: Geostatistical model of the spatial distribution of arsenic in groundwaters in Gujarat State, India
Source: Environ Geochem Health. 2020 Jul 11;43(7):2649–64. doi: 10.1007/s10653-020-00655-7 (PMC8275508; doi:10.1007/s10653-020-00655-7)
Supplement: Supplementary file 1 — Supplementary material 1 (DOCX 45 kb) [file 10653_2020_655_MOESM1_ESM.docx]

**Geostatistical model of the spatial distribution of arsenic in groundwaters in Gujarat State, India**

**Ruohan Wu ^1^, Joel Podgorski ^1,2^ , Michael Berg ^2^ and David A. Polya ^1,*^**

^1^ Department of Earth and Environmental Sciences, School of Natural Sciences & Williamson Research Centre for Molecular Environmental Science, University of Manchester, Manchester, M13 9PL, UNITED KINGDOM;

^2^ Eawag, Swiss Federal Institute of Aquatic Science and Technology, 8600 Dübendorf, SWITZERLAND;

***** Correspondence: david.polya@manchester.ac.uk (D.A.P.)

**Supplementary Materials**

8 Tables & 7 Figures

Table S1 Summary of attributes of independent variables in modelling: Abbreviations, units, types, resolution, and data sources

| NO. | Variables | Abbreviation | Unit | Type | Resolution | Data Sources |
| --- | --- | --- | --- | --- | --- | --- |
| 1 | Actual evapotranspiration | AET | mm/year | Continuous | 1km | Trabucco and Zomer, 2010 |
| 2 | Aridity |  | dimensionless | Continuous | 1km | Trabucco and Zomer, 2009 |
| 3 | Calcisols |  | % | Continuous | 250m | ISRIC, 2017 |
| 4 | Fluvisols |  | % | Continuous | 250m | ISRIC, 2017 |
| 5 | Gleysols |  | % | Continuous | 250m | ISRIC, 2017 |
| 6 | Potential evapotranspiration | PET | mm/year | Continuous | 1km | Trabucco and Zomer, 2009 |
| 7 | Precipitation |  | mm/year | Continuous | 1km | Hijmans et al., 2005 |
| 8 | Priestly-Taylor Alpha Coefficients | PTALCOE | dimensionless | Continuous | 1km | Trabucco and Zomer, 2010 |
| 9 | Slope |  | degree | Continuous | 1km | The World Bank, 2017 |
| 10 | Soil and sedimentary deposit thickness | ASSDT | meters | Continuous | 1km | Pelletier et al., 2016 |
| 11 | Soil cation exchange capacity | Soil CEC | meq / 100g clay | Continuous | 250m | ISRIC, 2017 |
| 12 | Soil organic carbon content | ORCDRC | k / kg | Continuous | 250m | ISRIC, 2017 |
| 13 | Soil organic carbon density | OCDENS | kg / m^3^ | Continuous | 250m | ISRIC, 2017 |
| 14 | Soil organic carbon stock | OCSTHA | ton/ha | Continuous | 250m | ISRIC, 2017 |
| 15 | Soil pH |  | dimensionless | Continuous | 250m | ISRIC, 2017 |
| 16 | Soil water capacity | WWP | % | Continuous | 250m | ISRIC, 2017 |
| 17 | Solonchaks |  | % | Continuous | 250m | ISRIC, 2017 |
| 18 | Temperature |  | $℃$ | Continuous | 1km | Hijmans et al., 2005 |
| 19 | Topographic wetness index | TWI | dimensionless | Continuous | 1km | Hengl, 2018 |
| 20 | Volume percentage of coarse fragments | CRFVOL | % | Continuous | 250m | ISRIC, 2017 |
| 21 | Water table depth | WTD | meters | Continuous | 1km | Fan et al., 2013 |
| 22 | Weight percentage of clay particles | CLYPPT | % | Continuous | 250m | ISRIC, 2017 |
| 23 | Weight percentage of sand particles | SNDPPT | % | Continuous | 250m | ISRIC, 2017 |
| 24 | Weight percentage of silt particles | SLTPPT | % | Continuous | 250m | ISRIC, 2017 |
| 25 | Acid plutonic rocks |  | dimensionless | Categorical | polygon | Hartmann & Moosforf, 2012 |
| 26 | Basic volcanic rocks |  | dimensionless | Categorical | polygon | Hartmann & Moosforf, 2012 |
| 27 | Metamorphic rocks |  | dimensionless | Categorical | polygon | Hartmann & Moosforf, 2012 |
| 28 | Sedimentary rocks |  | dimensionless | Categorical | polygon | Hartmann & Moosforf, 2012 |

Table S2 Parameter values for prevalence ratio of arsenic-induced skin cancer and incidence rate of arsenic-induced inner cancer. Estimated by Brown et al. (1989) and NRC (1999, 2001).

|  | $q_{1}$ | $q_{2}$ | $k$ | $m$ |
| --- | --- | --- | --- | --- |
|  | Skin Cancer | | | |
| Male | 7.936 x 10^-10^ | 1.640 x 10^-12^ | 2.950 | 6.873 |
| Female | 6.291 x 10^-11^ | 3.265 x 10^-13^ | 3.231 | 9.000 |
|  | Lung cancer | | | |
| Male | 1.4672 x 10^-11^ | 0 | 3.9195 | 21.4946 |
| Female | 0 | 6.1194 x 10^-14^ | 3.5137 | 17.0978 |
|  | Bladder cancer | | | |
| Male | 0 | 7.3394 x 10^-17^ | 5.1306 | 14.7025 |
| Female | 0 | 2.2225 x 10^-13^ | 3.4732 | 33.0365 |
|  | Liver cancer | | | |
| Male | 3.6947 x 10^-14^ | 4.9984 x 10^-13^ | 2.9054 | 16.8998 |
| Female | 2.8015 x 10^-8^ | 4.9395 x 10^-13^ | 2.7282 | 25.9420 |

Table S3 Independent variables appearing in logistic regressions using thresholds of 10 μg/L, 5 μg/L, 4 μg/L, 3μg/L, 2 μg/L, and 1 μg/L for groundwater arsenic and which passed the Hosmer-Lemeshow goodness-of-fit test. See Table S1 for explanation of variable abbreviations.

| Thresholds | Variables |
| --- | --- |
| 10 μg/L | aridity, PET, slope, WWP |
| 5 μg/L | aridity, ASSDT, CRFVOL, DTM, PET, fluvisols, slope, SNDPPT, soil_CEC, temperature, WWP |
| 4 μg/L | ASSDT, calcisols, CLYPPT, CRFVOL, DTM, PET, fluvisols, slope, sndppt, soil pH, WTD, WWP |
| 3 μg/L | aridity, CRFVOL, TWI, PET, fluvisols, slope, soil pH, temperature, WTD |
| 2 μg/L | aridity, CRFVOL, TWI, PET, slope, soil pH, temperature |
| 1 μg/L | evapotranspiration, temperature |

Table S4 Comparison of the goodness of fit of the logistic regression models for thresholds of 10 μg/L for groundwater arsenic with various variable combinations passing the Hosmer-Lemeshow goodness-of-fit test. Goodness of fit indicated by AUC values for modelling the entire dataset. Mean AUC and AIC values on testing datasets also tabulated. Coefficients of normalized variables give an indication of the relative importance of each variable within a given model. Preferred variable model as determined by highest AUC value indicated in bold.

| NO. | Variable Combinations (coefficients of normalized variables) | AUC | Mean AUC | Mean AIC |
| --- | --- | --- | --- | --- |
| 1 | slope (-23.90) | 0.7711 | 0.7681 | 131.5031 |
| 2 | aridity (-8.19), slope (-30.35) | 0.8092 | 0.8085 | 125.4225 |
| 3 | PET (3.55), slope (-23.72) | 0.8132 | 0.8089 | 123.7148 |
| 4 | slope (-23.26), WWP (-3.55) | 0.7999 | 0.7974 | 127.0251 |
| 5 | aridity (-10.81), slope (-31.12), WWP (-3.50) | 0.8175 | 0.8167 | 117.1626 |
| 6 | **PET** (3.47)**, slope** (-25.19)**, WWP** (-3.55) | **0.8275** | **0.8247** | **119.3607** |

Table S5 Comparison of the goodness of fit of the logistic regression models for thresholds of 5 μg/L for groundwater arsenic with various variable combinations passing the Hosmer-Lemeshow goodness-of-fit test. Goodness of fit indicated by AUC values for modelling the entire dataset. Mean AUC and AIC values on testing datasets also tabulated. Coefficients of normalized variables give an indication of the relative importance of each variable within a given model. Preferred variable model as determined by highest AUC value indicated in bold.

| NO. | Variable Combinations (coefficients of normalized variables) | AUC | Mean AUC | AIC |
| --- | --- | --- | --- | --- |
| 1 | aridity (-6.17), temperature (4.57) | 0.7688 | 0.7737 | 231.2336 |
| 2 | ASSDT (1.57), PET (3.14) | 0.7779 | 0.7822 | 224.0768 |
| 3 | TWI (6.76), PET (3.14) | 0.7589 | 0.7631 | 228.3559 |
| 4 | PET (3.99), fluvisols (3.22) | 0.7599 | 0.7628 | 228.1875 |
| 5 | PET (3.61), slope (-11.54) | 0.7746 | 0.7785 | 227.1861 |
| 6 | aridity (-5.32), fluvisols (2.84), temperature (4.40) | 0.7912 | 0.7956 | 223.9598 |
| 7 | **ASSDT** (1.18)**, PET** (2.67)**, slope** (-10.51) | **0.7937** | **0.7983** | **218.7494** |
| 8 | ASSDT (1.28), PET (3.35), Soil CEC (-2.54) | 0.7811 | 0.7858 | 220.1722 |
| 9 | CRFVOL (-2.82), PET (3.79), fluvisols (2.97) | 0.7841 | 0.7885 | 225.0644 |
| 10 | TWI (6.01), PET (3.18), Soil CEC (-2.78) | 0.7738 | 0.7787 | 224.2508 |
| 11 | TWI (6.03), PET (2.85), WWP (-2.27) | 0.7811 | 0.7848 | 225.3846 |
| 12 | TWI (5.26), SNDPPT (2.93), temperature (3.84) | 0.7758 | 0.7810 | 226.237 |
| 13 | PET (3.39), fluvisols (2.63), slope (-10.99) | 0.7910 | 0.7953 | 221.4558 |
| 14 | PET (3.84), fluvisols (3.01), Soil CEC (-2.58) | 0.7739 | 0.7791 | 222.9548 |
| 15 | PET (3.62), slope (-11.40), SNDPPT (2.88) | 0.7809 | 0.7858 | 223.6855 |
| 16 | PET (3.69), slope (-9.69), Soil CEC (-2.76) | 0.7853 | 0.7909 | 223.8178 |
| 17 | PET (3.38), slope (-10.28), WWP (-2.45) | 0.7930 | 0.7975 | 222.6845 |

Table S6 Comparison of the goodness of fit of the logistic regression models for thresholds of 4 μg/L for groundwater arsenic with various variable combinations passing the Hosmer-Lemeshow goodness-of-fit test. Goodness of fit indicated by AUC values for modelling the entire dataset. Mean AUC and AIC values on testing datasets also tabulated. Coefficients of normalized variables give an indication of the relative importance of each variable within a given model. Preferred variable model as determined by highest AUC value indicated in bold.

| NO. | Variable Combinations (coefficients of normalized variables) | AUC | Mean AUC | AIC |
| --- | --- | --- | --- | --- |
| 1 | ASSDT (1.09), TWI (5.24) | 0.7277 | 0.7263 | 279.3212 |
| 2 | ASSDT (1.36), PET (2.79) | 0.7435 | 0.7405 | 277.1803 |
| 3 | calcisols (2.56), TWI (8.26) | 0.7210 | 0.7208 | 279.4668 |
| 4 | TWI (6.06), PET (2.67) | 0.7400 | 0.7393 | 280.2533 |
| 5 | TWI (5.27), fluvisols (2.14) | 0.7289 | 0.7299 | 291.3026 |
| 6 | PET (3.50), fluvisols (3.30) | 0.7487 | 0.7483 | 278.7497 |
| 7 | PET (3.02), slope (-9.15) | 0.7438 | 0.7427 | 280.8296 |
| 8 | ASSDT (1.15), CRFVOL (-2.38), PET (2.31) | 0.7457 | 0.7427 | 276.9598 |
| 9 | ASSDT (0.10), TWI (4.86), PET (2.18) | 0.7541 | 0.7522 | 271.9397 |
| 10 | ASSDT (1.02), PET (2.78), fluvisols (2.52) | 0.7638 | 0.7622 | 272.8171 |
| 11 | ASSDT (1.01), PET (2.44), slope (-7.10) | 0.7573 | 0.7553 | 273.4258 |
| 12 | calcisols (2.06), TWI (6.66), PET (2.05) | 0.7493 | 0.7496 | 277.181 |
| 13 | CRFVOL (-2.69), PET (3.11), fluvisols (3.45) | 0.7670 | 0.7661 | 273.7767 |
| 14 | **TWI** (4.83)**, PET** (2.76)**, fluvisols** (2.58) | **0.7680** | **0.7680** | **274.4554** |
| 15 | TWI (5.72), PET (2.39), SNDPPT (2.50) | 0.7444 | 0.7451 | 276.1734 |
| 16 | TWI (5.50), PET (2.71), Soil CEC (-2.20) | 0.7449 | 0.7456 | 276.887 |
| 17 | TWI (6.65), PET (3.59), Soil pH (-3.37) | 0.7380 | 0.7386 | 276.1097 |
| 18 | TWI (5.58), PET (2.96), WWP (-1.79) | 0.7486 | 0.7492 | 272.4696 |
| 19 | PET (3.46), fluvisols (3.07), slope (2.53) | 0.7653 | 0.7660 | 273.9498 |
| 20 | PET (3.45), fluvisols (2.92), WTD (-4.36) | 0.7619 | 0.7614 | 270.9427 |
| 21 | PET (2.81), slope (-9.66), SNDPPT (2.54) | 0.7540 | 0.7544 | 277.0629 |
| 22 | ASSDT (1.56), CLYPPT (4.95), PET (2.90), sndppt (4.99) | 0.7524 | 0.7518 | 274.8691 |
| 23 | calcisols (2.19), TWI (7.01), PET (3.27), Soil pH (-3.68) | 0.7530 | 0.7440 | 272.4391 |
| 24 | TWI (6.16), PET (3.16), SNDPPT (2.26), Soil pH (-3.28) | 0.7465 | 0.7471 | 277.8261 |

Table S7 Comparison of the goodness of fit of the logistic regression models for thresholds of 3 μg/L for groundwater arsenic with various variable combinations passing the Hosmer-Lemeshow goodness-of-fit test. Goodness of fit indicated by AUC values for modelling the entire dataset. Mean AUC and AIC values on testing datasets also tabulated. Coefficients of normalized variables give an indication of the relative importance of each variable within a given model. Preferred variable model as determined by highest AUC value indicated in bold.

| NO. | Variable Combinations (coefficients of normalized variables) | AUC | Mean AUC | AIC |
| --- | --- | --- | --- | --- |
| 1 | aridity (-4.98), TWI (3.97) | 0.6981 | 0.6977 | 358.9264 |
| 2 | aridity (-6.13), slope (-7.89) | 0.7143 | 0.7143 | 352.2671 |
| 3 | aridity (-7.97), temperature (3.08) | 0.7487 | 0.7491 | 347.5423 |
| 4 | TWI (4.36), PET (2.93) | 0.7166 | 0.7167 | 351.5099 |
| 5 | PET (4.19), fluvisols (2.68) | 0.7161 | 0.7159 | 341.7448 |
| 6 | PET (3.20), slope (-5.93) | 0.7167 | 0.7162 | 353.2016 |
| 7 | aridity (-6.53), TWI (3.59), temperature (2.18) | 0.7456 | 0.7457 | 344.3414 |
| 8 | aridity (-4.27), PET (2.15), slope (-6.28) | 0.7363 | 0.7364 | 346.9731 |
| 9 | **aridity** (-6.99)**, fluvisols** (1.97)**, temperature** (2.94) | **0.7575** | **0.7675** | **344.3026** |
| 10 | aridity (-7.31), slope (-5.54), temperature (2.27) | 0.7547 | 0.7550 | 342.7834 |
| 11 | aridity (5.25), Soil pH (3.97), temperature (-3.24) | 0.7574 | 0.7575 | 342.0068 |
| 12 | TWI (3.41), PET (2.24), Soil pH (-4.86) | 0.7162 | 0.7163 | 346.5254 |
| 13 | PET (3.41), fluvisols (2.24), slope (-4.86) | 0.7283 | 0.7282 | 346.3579 |
| 14 | aridity (-7.24), CRFVOL (-2.32), PET (3.49), Soil pH (-3.90) | 0.7373 | 0.7372 | 346.5548 |
| 15 | aridity (-5.13), TWI (4.25), PET (3.05), Soil pH (-3.79) | 0.7419 | 0.7418 | 344.4279 |
| 16 | aridity (-8.69), TWI (3.51), Soil pH (-3.28), temperature (2.27) | 0.7559 | 0.7559 | 341.8046 |
| 17 | aridity (-6.06), PET (2.88), slope (-6.16), Soil pH (-3.41) | 0.7426 | 0.7424 | 342.9930 |

Table S8 Comparison of the goodness of fit of the logistic regression models for thresholds of 2 μg/L for groundwater arsenic with various variable combinations passing the Hosmer-Lemeshow goodness-of-fit test. Goodness of fit indicated by AUC values for modelling the entire dataset. Mean AUC and AIC values on testing datasets also tabulated. Coefficients of normalized variables give an indication of the relative importance of each variable within a given model. Preferred variable model as determined by highest AUC value indicated in bold.

| NO. | Variable Combinations (coefficients of normalized variables) | AUC | Mean AUC | AIC |
| --- | --- | --- | --- | --- |
| 1 | aridity (-5.31) | 0.6622 | 0.6630 | 409.9315 |
| 2 | PET (3.27) | 0.6756 | 0.6759 | 409.3184 |
| 3 | aridity (-4.77), CRFVOL (-2.13) | 0.6825 | 0.6811 | 407.2433 |
| 4 | aridity (-4.38), TWI (3.00) | 0.6713 | 0.6718 | 402.4405 |
| 5 | aridity (-3.40), PET (2.09) | 0.6988 | 0.6989 | 402.8651 |
| 6 | aridity (-4.78), slope (-3.46) | 0.6774 | 0.6781 | 400.3215 |
| 7 | **aridity (-5.99), temperature (1.95)** | **0.7069** | **0.7087** | **404.6537** |
| 8 | TWI (2.70), PET (2.66) | 0.6894 | 0.6870 | 400.5975 |
| 9 | PET (2.83), slope (-3.21) | 0.6869 | 0.6870 | 403.6573 |
| 10 | aridity (-3.21), PET (1.92), slope (-3.29) | 0.7034 | 0.7038 | 396.3125 |
| 11 | aridity (-5.28), PET (2.78), Soil pH (-2.84) | 0.6889 | 0.6892 | 399.0279 |
| 12 | aridity (-4.15), CRFVOL (-1.98), PET (2.74), Soil pH (-2.79) | 0.6980 | 0.6984 | 400.6386 |
| 13 | aridity (-4.81), PET (2.36), slope (-3.11), Soil pH (-2.96) | 0.6921 | 0.6913 | 399.2147 |

**Figure Captions – Supplementary Materials**

Fig. S1 Frequency diagram of Gujarat groundwater arsenic concentrations (n = 398; CGWB, 2016) used in the modelling.

Fig. S2 ROC curves of final logistic regression models with (a) 10 μg/L, (b) 5 μg/L, (c) 4 μg/L, (d) 3μg/L, (e) 2 μg/L, and (f) 1 μg/L as thresholds for groundwater arsenic in Gujarat state, India.

Fig. S3 Plots of sensitivity (true-positive rate), specificity (true-negative rate) and accuracy against cutoffs of the final logistic regression models with (a) 10 μg/L, (b) 5 μg/L, (c) 4 μg/L, (d) 3μg/L, and (e) 2 μg/L as thresholds for groundwater arsenic in Gujarat state, India.

Fig. S4 Hazard maps showing the probability of the geospatially modelled occurrences of groundwater arsenic concentration exceeding thresholds of (a) 10 μg/L, (b) 5 μg/L, (c) 4 μg/L, (d) 3μg/L, and (e) 2 μg/L in Gujarat state, India.

Fig. S5 Spatial distribution of predictor variables in the final logistic regression models of groundwater arsenic hazard distribution in Gujarat.

Fig. S6 Pseudo-contour map of geospatially modelled groundwater arsenic hazard distribution in Gujarat using a cut-off value (0.5) for all concentrations.

Fig. S7 Spatial distribution of soil organic carbon content at 1 – 2 m depth in Gujarat. (ISRIC, 2017)
