# Supplementary figures and images for: Geostatistical model of the spatial distribution of arsenic in groundwaters in Gujarat State, India
Source: Environ Geochem Health. 2020 Jul 11;43(7):2649–64. doi: 10.1007/s10653-020-00655-7 (PMC8275508; doi:10.1007/s10653-020-00655-7)

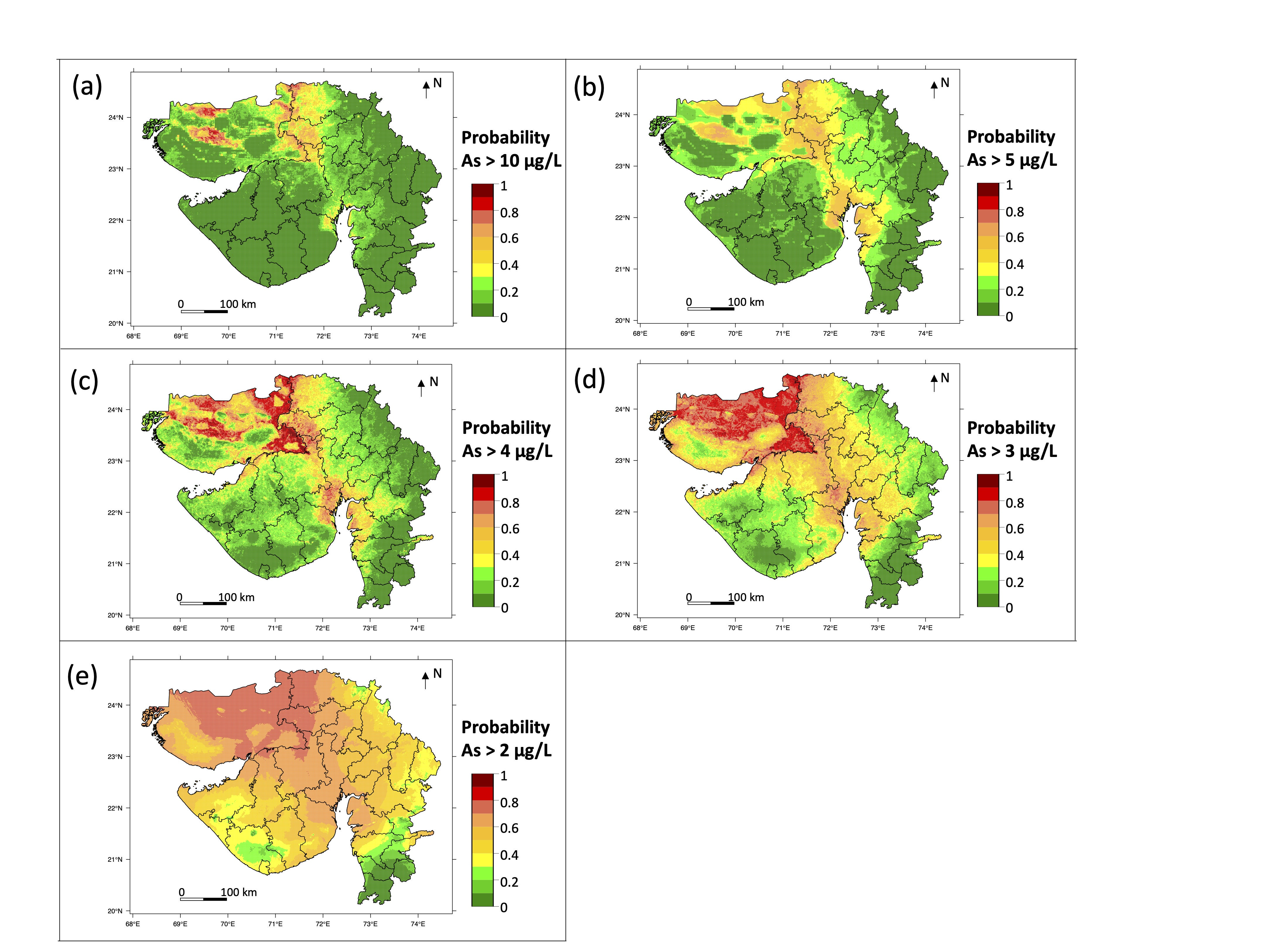

Supplement: Supplementary file 5 — Supplementary material 5 (TIFF 9625 kb) [file 10653_2020_655_MOESM5_ESM.tif]
